# Identification and validation of HELLS (Helicase, Lymphoid-Specific) and ICAM1 (Intercellular adhesion molecule 1) as potential diagnostic biomarkers of lung cancer

**DOI:** 10.7717/peerj.8731

**Published:** 2020-03-09

**Authors:** Wei Zhu, Lin Lin Li, Yiyan Songyang, Zhan Shi, Dejia Li

**Affiliations:** 1 Department of Occupational and Environmental Health, Wuhan University, School of Health Science, Wuhan, Hubei, China; 2 Human Biology Program, University of Toronto, Toronto, ON, Canada

**Keywords:** Lung cancer, Bioinformatics analysis, Prognosis, HELLS, ICAM1

## Abstract

Although lung cancer is one of the greatest threats to human health, its signaling pathway and related genes are still unknown. This study integrates data from three groups of people to study potential key candidate genes and pathways related to lung cancer. Expression profiles (GSE18842, GSE19188 and GSE27262), including 162 tumor tissue and 135 adjacent normal lung tissue samples, were integrated and analyzed. Differentially expressed genes (DEGs) and candidate genes were identified, their expression pathways were analyzed, and the diethylene glycol-related protein–protein interaction (PPI) network was analyzed. We identified 232 shared DEGs (40 upregulated and 192 down-regulated) from the three GSE datasets. The DEGs were clustered according to function and signaling pathway for significant enrichment analysis. In total, 129 nodes/DEGs were identified from the DEG PPI network complex. An improved prognosis was associated with increased Helicase, Lymphoid-Specific (HELLS) and decreased Intercellular adhesion molecule 1 (ICAM1) mRNA expression in lung cancer patients. In conclusion, we used integrated bioinformatics analysis to identify candidate genes and pathways in lung cancer to show that HELLS and ICAM1 might be the key genes related to tumorigenesis or tumor progression in lung cancer. Additional studies are needed to further explore the involved functional mechanisms.

## Introduction

Lung cancer is one of the most leading causes of cancer-related deaths worldwide. In 2016, there were more than 220,000 diagnoses and nearly 158,000 deaths from lung cancer in the United States alone (Siegel, Miller & Jemal, 2016). There are two main histological types of lung cancer: non-small cell lung cancer (NSCLC) and small cell lung cancer. The former accounts for about 85% of all lung cancer, including squamous cell carcinoma, adenocarcinoma and large cell carcinoma (Siegel, Miller & Jemal, 2018). Although significant progress has been made in diagnosis and treatment methods in the last 5 years, the overall survival (OS) rate of lung cancer is still less than 15% (Bironzo, Passiglia & Novello, 2019). Therefore, the molecular mechanisms involved in the development of lung cancer should be studied and clarified in order to improve survival rates.

Gene chip, or gene expression profile, is a genetic detection technology that is particularly useful for screening differential gene expression since it has the ability to rapidly detect all genes expressed in the same sample after the time of sampling (Vogelstein et al., 2013). The widespread use of gene chips has generated a large amount of core slice data stored in public databases. Useful information has been provided from integrating and analyzing this data. In recent years, a large number of microarray data analyses on lung cancer have been conducted, and hundreds of differentially expressed genes (DEGs) have been identified. However, due to the heterogeneity of the tissue or samples in the existing studies, the results were limited or inconsistent. In cancer tissues, heterogeneity means that cells with different gene mutations may have different biological characteristics. The clinical diagnosis of cancer by pathologists usually relies on limited samples of cancer tissue that do not represent heterogeneity, either between or within patients (Bedard et al., 2013; De Sousa & Carvalho, 2018).

As a result, reliable biomarkers have not been found in lung cancer. A novel approach to address these shortcomings is to incorporate a comprehensive bioinformatics approach to expression profiling techniques, which is the approach we adopted in this study.

We first downloaded three original microarray datasets, GSE18842 (Sanchez-Palencia et al., 2011), GSE19188 (Hou et al., 2010) and GSE27262 (Wei et al., 2014, 2012), from the NCBI Gene Expression Synthesis Database (NCBI–GEO, https://www.ncbi.nlm.nih.gov/geo) (Barrett et al., 2005). Data were obtained from 162 lung cancer cases and 135 adjacent normal tissues. The principles of our dataset selection were as following: (1) the sample size was greater than 50; (2) the samples were all from lung cancer patients and paracancer tissues; (3) these patients had not undergone any other drug intervention; and (4) the purposes of carrying out the gene chip or RNA-seq were to compare and analyze the RNA expression differences between lung cancer patients and paracancer tissues. We screened the corresponding DEGs according to the data processing standards of the Morpheus website and used DAVID, Cytoscape, Metascape (http://metascape.org/) (Zhou et al., 2019), UCSC (https://genome.ucsc.edu/) (Haeussler et al., 2019), cBioportal (Gao et al., 2013), BioCyc (http://biocyc.org) (Latendresse, Paley & Karp, 2012) and Panther (http://www.pantherdb.org) to perform gene ontology and pathway enrichment analysis. We also developed a comprehensive DEG protein–protein interaction (PPI) network and module analysis to identify the central gene of lung cancer using the Search Tool for the Retrieval of Interacting Genes/Proteins database (STRING, http://string-db.org). To identify the central lung cancer genes using string (http://string-db.org), we also developed a comprehensive DEG PPI network and module analysis. Helicase, Lymphoid-Specific (HELLS) and Intercellular adhesion molecule 1 (ICAM1) were identified, and their biological functions and key pathways were enriched to ascertain more accurate and practical biomarkers for the early diagnosis, individualized prevention, and treatment of lung cancer. Finally, we analyzed the expression of HELLS and ICAM1 in lung cancer patients to determine their expression patterns, potential functions, and different prognostic values.

## Materials and Methods

### Microarray data analysis and identification of DEGs

We obtained lung cancer and adjacent tissue gene expression profiles for GSE18842, GSE19188 and GSE27262 from NCBI to GEO, which is a free microarray/gene database repository of high throughput gene expression data. Microarray data for GSE18842 were based on the GPL570 platform ((HG-U133_Plus_2) Affymetrix Human Genome U133 Plus 2.0 Array), and included 46 tumors and 45 controls (submission date: 2 November 2009) (Latendresse, Paley & Karp, 2012). GSE19188 data were based on the GPL570 platform ((HG-U133_Plus_2) Affymetrix Human Genome U133 Plus 2.0 Array), and included 91 tumor and 65 adjacent normal lung tissue samples (submission date: 25 November 2009) (Hou et al., 2010). GSE27262 data were based on the GPL570 platform ((HG-U133_Plus_2) Affymetrix Human Genome U133 Plus 2.0 Array), and included 25 pairs of tumor and adjacent normal tissues from LUAD patients (submission date: 11 February 2011) (Wei et al., 2014, 2012). We selected these three datasets for integrated analysis and identified DEGs using a classical *t* test. The adjusted *p* values (adj. *p*) were utilized to correct the occurrence of false-positive results using the Benjamini and Hochberg false discovery rate method by default. In the present study, statistically significant DEGs were defined using values of adj. *p* < 0.05 and [logFC] > 1 as cutoff criteria (The Gene Ontology Consortium, 2015; Ashburner et al., 2000; Lebrec et al., 2009).

### Gene ontology and pathway enrichment analysis

Metascape (http://metascape.org/) is an online analysis tool for extracting comprehensive biometric information from huge lists of candidate genes. It not only performs typical genetic terminology enrichment analysis, but also visualizes the relationship between genomic terms, searches for interesting and related genes or terms, and dynamically views genes from their biological functions and pathways. GO analysis and the Kyoto Genomics and Genomics Encyclopedia (KEGG) path analysis were conducted on the selected DEG using the Metascape tool. The enrichment score [−log 10 (*p*-value)] was significantly ranked with a *p*-value <0.01 as the cutoff criterion.

### Integration of PPI network complex identification

We developed a DEG-encoded protein and PPI network using STRING (http://string-db.org) (Szklarczyk et al., 2019). The PPI network was constructed using Cytoscape software (version 3.7.1) to analyze the interactions between candidate DEG-encoded proteins in lung cancer (Kohl, Wiese & Warscheid, 2011). The Node Analyzer was calculated using the Network Analyzer plug-in, which reveals the number of connections used to filter the PPI hub genes. The corresponding protein identified at the central node may be a core protein and key candidate gene with important physiological regulatory functions.

### Identification and clinical significance of central genes

Hub genes were identified using CythopCAE’s CyoHubBA toolkit. The top 10 central genes with less than 10 degrees were selected. Hierarchical clustering of hub genes in the Cancer Genome Atlas (TCGA) database was constructed using the UCSC Cancer Genome Browser (https://genome.ucsc.edu/). Biological process analysis of the hub gene was performed utilizing the Cytoscape Bionetwork Gene Oncology Tool (BiNGO) plug-in. The frequency of gene changes was assessed using the cBioportal online database (http://www.cbioportal.org/). PPI networks were built using STRING.

### Prognosis analysis using Kaplan–Meier plots

The TCGA online database, which contains gene expression data and survival information for lung cancer patients and sequencing and pathological data for 30 different cancers, was utilized to evaluate the prognostic value of DEG expression (The Cancer Genome Atlas Network, 2012). To analyze the OS of patients with lung cancer, 545 patients were assigned to either two groups (high and low expression) or three groups (high, medium and low expression) by median expression and were assessed using Kaplan–Meier survival analysis with hazard ratio, 95% confidence intervals, and log-rank *p*-value. Only the JetSet best probe set of DEGs was selected to obtain a Kaplan–Meier plot, with the risk number shown below the main plot.

### Lung cancer samples

Lung cancer and adjacent normal tissues were obtained after surgical resection of patients with NSCLC being treated at Wuhan University Affiliated Hospital, Hubei Provincial People’s Hospital, and Zhongnan Hospital of Wuhan University, China. Informed consent was received by each patient. The study was approved by Wuhan University’s Institute of Ethics with certificate number 2018001. It is presumed that informed consent had been obtained for all datasets used from the published literature.

### RT-qPCR

Total RNA was extracted from lung cancer cells and tissues using Trizol reagent (Invitrogen; Thermo Fisher Scientific, Inc., Waltham, MA, USA). A reverse transcription kit (Vazyme Biotech Co., China) was used to reverse transcribe RNA and mRNA expression was evaluated using the 2^(ΔΔCt)^ method. Relative gene expression was then calculated and normalized to endogenous glyceraldehyde 3-phosphate dehydrogenase (GAPDH). The primers of GAPDH, HELLS, ICAM1 were purchased from Qingke Biotechnology Co., Ltd., China. The forward and reverse primer sequences are as follows: GAPDH F: 5′-CCTTCCGTGTCCCCACT-3′ and GAPDH R: 5′-GCCTGCTTCACCACCTTC-3′, HELLS F: 5′-CCCTCCTTTCTTCTAGTAATGCAGTT-3′ and HELLS R: 5′-CCCAATCTCTCCCCATGAAAA-3′; ICAM1 F: 5′-GAACCCATTGCCCGAGCTCA-3′ and ICAM1 R: 5′-TGACAGTCACTGATTCCCCGAT-3′.

### Cell culture

Human NSCLC cell lines A549, H1299, PC9 and HCC827 and the normal lung epithelial cell line BEAS2B were all bought from the Shanghai Cell Bank of the Chinese Academy of Sciences and were cryopreserved in liquid nitrogen tanks. The cells were cultured in DubCo’s MudieEdEdE medium (Hyo Corporation, Salt Lake City, UT, USA) with 10% fetal bovine serum (GiBCO Co., Grand Island, NY, USA) and incubated at 37 °C in a 5% CO2 atmosphere.

### Statistical analysis

Experimental data were recorded in Excel and analyzed using GraphPad Prism 7. The results were analyzed by a two-tailed *t* test. Values of *p* < 0.05 were considered significant differences. Data were expressed as mean ± standard error.

## Results

### Identification of DEGs in lung cancer

**Figure 1:**
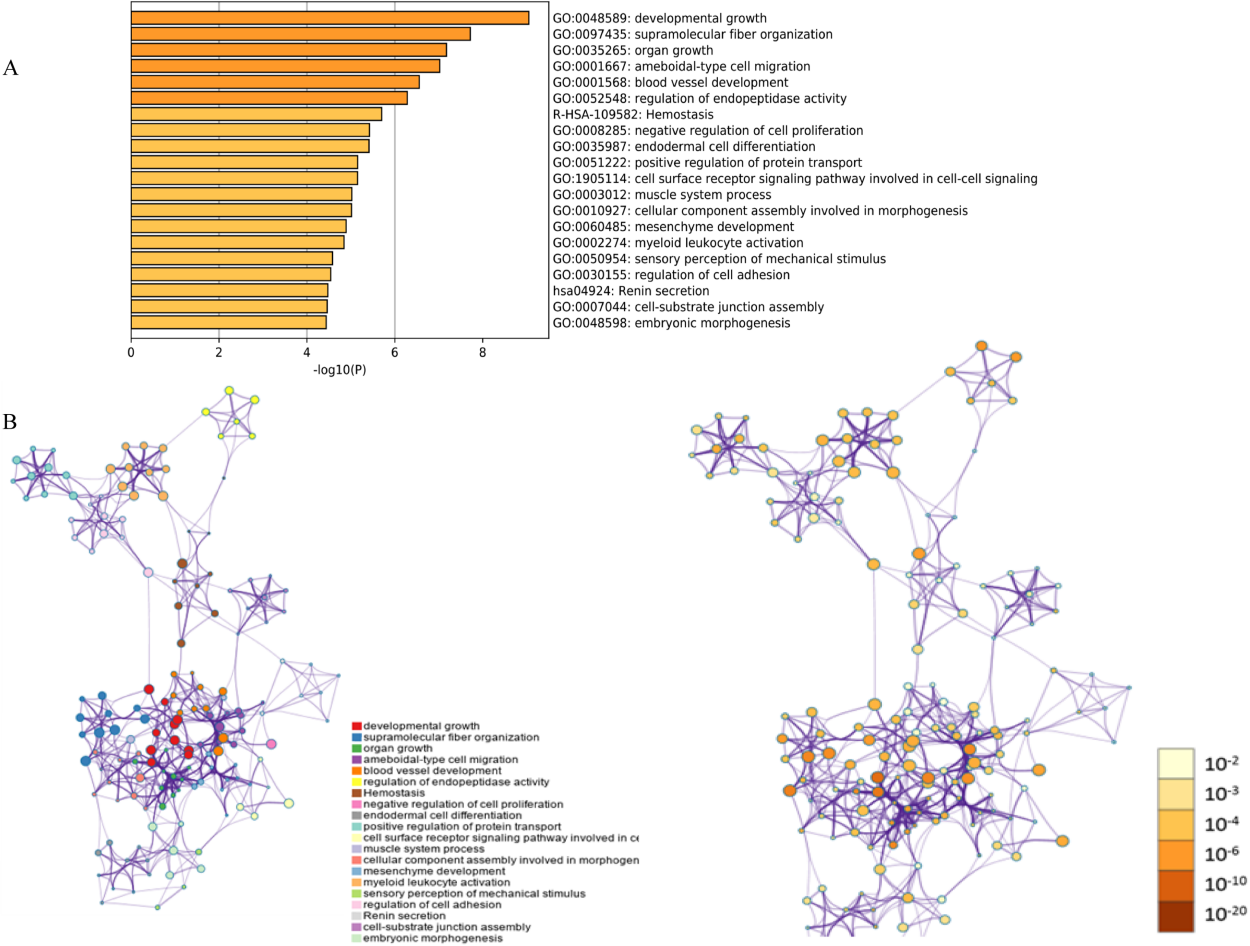
Functional and pathway enrichment analysis of DEGs. (A) GO terms and KEGG pathway were presented, and each band represents one enriched term or pathway colored according to the −log 10 *p* value. (B) Network of the enriched terms and pathways. Nodes represent enriched terms or pathways with node size indicating the number of DEGs involved in. Nodes sharing the same cluster are typically close to each other, and the thicker the edge displayed, the higher the similarity is represented.

**Figure 2:**
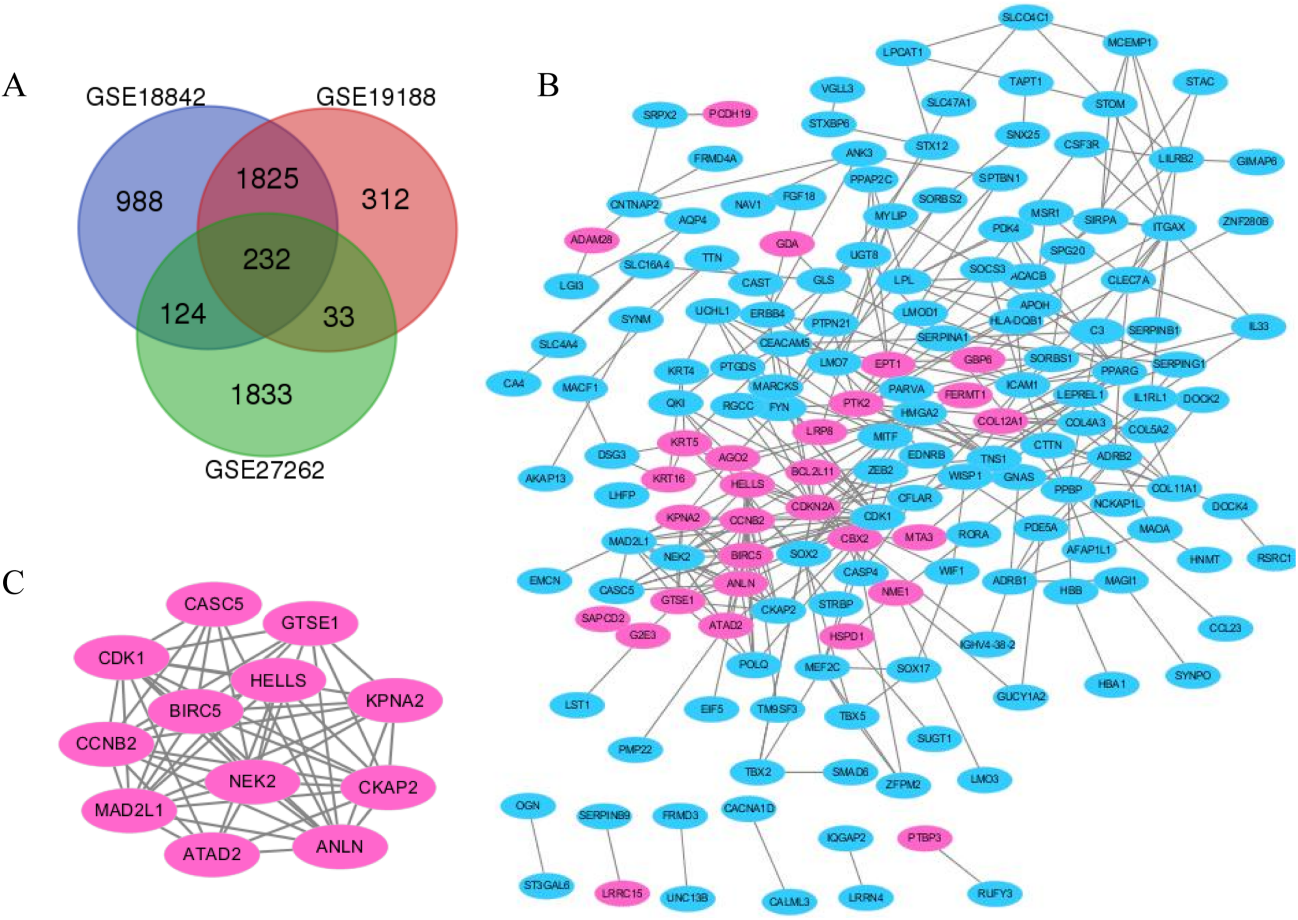
The distribution of differential genes between GSE18842, GSE19188 and GSE27262. (A) DEGs were selected with *p* < 0.05 and [log FC] > 1 among the mRNA expression profiling sets. (B and C) The PPI network of DEGs was constructed using Cytoscape (upregulated genes are marked in light red; downregulated genes are marked in light blue).

Lung cancer and adjacent normal tissue gene expression profiles for GSE18842, GSE19188 and GSE27262 were obtained from NCBI–GEO. Microarray data for GSE18842 comprised 46 tumors and 45 controls (Sanchez-Palencia et al., 2011). GSE21815 data comprised 91 tumor and 65 adjacent normal lung tissue samples. GSE27262 data included 25 paired tumor and adjacent normal tissue samples. In total, 2,042, 1,424 and 1,142 DEGs were extracted from the GSE18842, GSE19188 and GSE27262 expression profile datasets, respectively, using adj. *p* < 0.05 and [logFC] > 1 as cutoff criteria. A total of 232 uniformly expressed genes were identified from the three profile datasets using integrated analysis (Figs. 1 and 2). When compared to normal lung tissue, lung cancer tissues included 40 up-regulated genes and 192 down-regulated genes (Table 1).

**Table 1:** Two hundred thirty-two differentially expressed genes (DEGs) were screened from three profile datasets.

DEGs	Genes symbol
Upregulated (40)	G2E3, GDA, EPT1, KPNA2, IGH, ANLN, BIRC5, HSPD1, PTK2, BC017398, MIR3934, SAPCD2, IGHD, AGO2, GTSE1, CBX2, PTBP3, ADAM28, CCNB2, LRP8, NFE2L3, KRT16, IGHG1, NME1, COL12A1, EYA2, LEPREL4, LRRC15, BCL2L11, ATAD2, MTA3, HIST1H2BG, PCDH19, SLC1A4, HELLS, GBP6, FERMT1, KRT5, HOXC6, CDKN2A
Downregulated (192)	PFKP, ERG, HIGD1B, VGLL3, IPW, MAOA, CDK1, RSRC1, SPTBN1, SNX25, UNC13B, PPBP, QKI, SPG20, MAD2L1, SORBS2, CCM2L, WIF1, GIMAP6, SOCS3, MAGI1, LMO7, CCL23, PCDP1, OGN, KRT4, SFTA3, CEACAM5, PDE5A, SLC16A4, PDZD2, WISP1, HBB, ITGAX, TM9SF3, LPL, COL11A1, ODF3B, CASP4, ROR1, SYNM, UGT8, FKBP11, SESTD1, SLC4A4, RP699M1.2, CTTN, NEK2, SMAD6, MEF2C, ERBB4, RP115C23.1, MACF1, MAGEA10, MAGEA5, ITIH5, SIGLEC17P, TBX5, PARVA, PPAP2C, AQP4, SLC47A1, SERPINA1, COL4A3, IL1RL1, MCEMP1, CYP4V2, TRPV2, STOM, KIAA1244, EDNRB, ST3GAL6, SOX17, TNS1, CAMK2N1, POLQ, CACNA1D, RGS5, PTGDS, GAGE12B, EIF5, SERPINB1, PTPN21, CST6, STRBP, NAV1, SHROOM3, CNTNAP2, ZNF280B, LMO3, MBIP, IL33, ARHGAP6, RNF125, CYP2B6, ANK3, DAPL1, KCTD1, TACC2, MITF, LILRB2, HOXA1, CSF3R, LOC643733, HNMT, GNAS, SLC27A3, SERPINB9, C3, SERPING1, AFAP1L1, SULT1A2, ZFPM2, SEC63, ADRB1, SVEP1, FYN, COL5A2, LOC101928198, PLAC9, MSR1, LST1, DOCK4, FRMD4A, KLF9, PDK4, EMCN, TSPAN12, CA4, SRPX2, SIRPA, APOH, CLEC7A, NCKAP1L, LHFP, GLS, CFLAR, ACACB, RUFY3, SOBP, PMP22, P2RX7, LEPREL1, LPCAT1, SOX2, IQGAP2, OTUD1, FRMD3, DOCK2, BTNL9, UCHL1, CLEC2B, TBX2, TMEM237, PPARG, HLADQB1, LMOD1, SUGT1, LRRN4, RGCC, ADRB2, CMAHP, SEMA6A, HMGA2, CCDC68, SREK1IP1, MYLIP, DOCK9, MARCKS, RORA, SORBS1, GUCY1A2, STXBP6, STX12, CASC5, CALML3, CKAP2, ICAM1, FGF18, ZEB2, DSG3, LGI3, TTN, AKAP13, SLC34A2, STAC, TAPT1, SEMA5A, SYNPO, CAST, SLCO4C1, HBA1

### Functional and pathway enrichment analysis of DEGs

The functions and pathways of the candidate DEGs were predicted using the Metascape Database (Table 2) (Geiman, Durum & Muegge, 1998). There were 18 terms and two pathways involved in the DEGs enrichment analysis (Fig. 1A), and the DEGs were mainly enriched during developmental growth, embryonic morphogenesis, cell-substrate junction assembly, renin secretion, regulation of cell adhesion, myeloid leukocyte activation, mesenchyme development, assembly of cellular components involved in morphogenesis and muscle system processes, in the cell surface receptor signaling pathway involved in cell–cell signaling, positive regulation of protein transport, endodermal cell differentiation, negative regulation of cell proliferation, hemostasis and ameboidal-type cell migration. The GO function and KEGG pathway enrichment analysis of candidate DEGs are shown in Fig. 1. The enriched terms were closely connected with each other and clustered into intact networks (Fig. 1B). These results indicate that most DEGs are significantly enriched during cardiomyocyte proliferation, protein binding, and the positive regulation of cell membranes.

**Table 2:** Pathway and process enrichment analysis.

GO	Category	Description	Count	%	log 10 (*p*)
Upregulated					
M236	Canonical Pathways	PID DELTA NP63 PATHWAY	4	10.53	−6.05
M176	Canonical Pathways	PID FOXM1 PATHWAY	3	7.89	−4.47
R-HSA-5693606	Reactome Gene sets	DNA Double Strand Break Response	3	7.89	−3.62
M66	Canonical Pathways	PID MYC ACTIV PATHWAY	4	7.89	−3.58
GO:0007160	GO Biological Processes	Ell-matrix adhesion	5	10.53	−3.37
GO:1903828	GO Biological Processes	Negative regulation of cellular protein localization	3	7.89	−3.16
GO:0008637	GO Biological Processes	Apoptotic mitochondrial changes	3	7.89	−3.01
R-HSA-9006925	Reactome Gene sets	Intracellular signaling by second messenger	3	10.53	−2.89
GO:0042742	GO Biological Processes	Defense response to bacterium	4	10.53	−2.78
GO:0051301	GO Biological Processes	Cell division	5	13.16	−2.65
GO:0017038	GO Biological Processes	Protein import	3	7.89	−2.48
GO:0048872	GO Biological Processes	Homeostasis of number of cells	3	7.89	−2.15
Downregulated					
GO:0048589	GO Biological Processes	Developmental growth	23	12.17	−8.59
GO:0030036	GO Biological Processes	Actin cytoskeleton organization	22	11.64	−8.04
GO:0001568	GO Biological Processes	Blood vessel development	22	11.64	−6.79
GO:0002274	GO Biological Processes	Myeloid leukocyte activation	19	10.05	−6.01
GO:0052548	GO Biological Processes	Regulation of endopeptidase activity	15	7.94	−5.93
GO:0060485	GO Biological Processes	Mesenchyme development	12	6.35	−5.70
R-HSA-109582	Reactome Gene sets	Hemostasis	18	9.52	−5.67
GO:0010927	GO Biological Processes	Cellular component assembly involved in morphogenesis	8	4.23	−5.60
GO:0003012	GO Biological Processes	Muscle system process	15	7.94	−5.36
GO:1905114	GO Biological Processes	Cell surface receptor signaling pathway involved in cell–cell signaling	17	8.99	−5.08
GO:0008285	GO Biological Processes	Negative regulation of cell proliferation	19	10.05	−5.00
hsa04924	KEGG Pathway	Renin secretion	6	3.17	−4.92
GO:0048588	GO Biological Processes	Developmental cell growth	10	5.29	−4.86
GO:0010884	GO Biological Processes	Positive regulation of lipid storage	4	2.12	−4.62
GO:0003013	GO Biological Processes	Circulatory system process	15	7.94	−4.55
GO:0051345	Reactome Gene Sets	Posithive regulation of hydrolase activity	18	9.52	−4.49
GO:0043408	GO Biological Processes	Regulation of MAPK cascade	18	9.52	−4.44
R-HSA-1247673	GO Biological Processes	Erythrocytes take up oxygen release	3	1.59	−4.42
GO:0099612	GO Biological Processes	Protein localization to axon	3	1.59	−4.42
GO:0045444	GO Biological Processes	Fat cell differentiation	9	4.76	−4.21

### DEGs modular analysis with PPI network and hub gene identification

**Figure 3:**
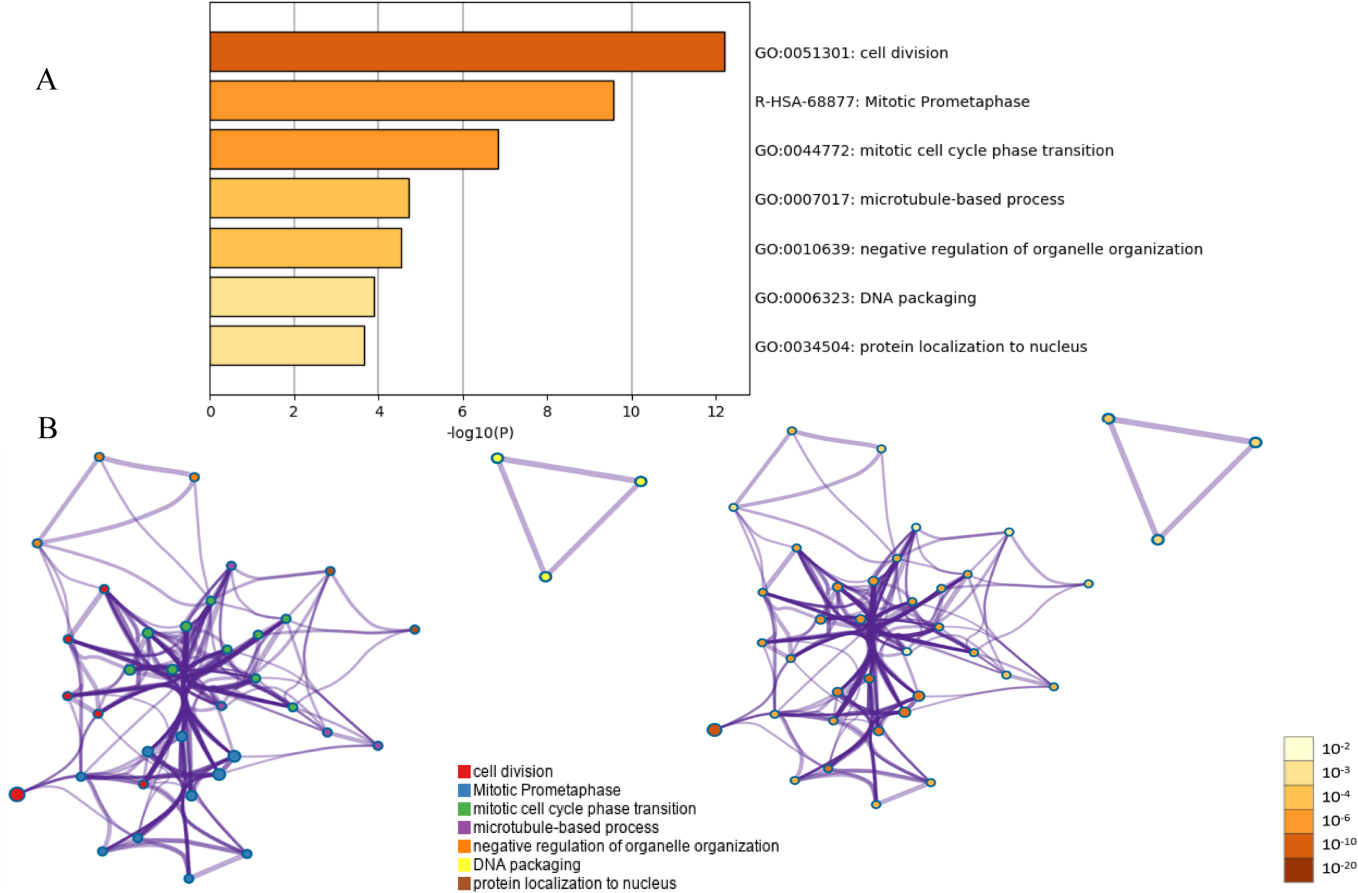
Functional and pathway enrichment analysis of the PPI module. (A) GO terms and KEGG pathway were presented, and each band represents one enriched term or pathway colored according to the −log 10 *p* value. (B) Network of the enriched terms and pathways. Nodes represent enriched terms or pathways with node size indicating the number of DEGs involved in.

Protein interaction networks have proven to be powerful tools for predicting new essential genes in specific signal transduction pathways. Using the STRING online database and Cytoscape software 13, a total of 232 DEGs were filtered into the PPI network complex. The PPI network of DEGs was constructed with the most important module obtained using the Cytoscape (Figs. 2B and 2C). Using the Metascope to analyze the functions of the genes involved in this module, we found that the functions mainly focused on cell division and pre-mitotic stage and mitosis cell cycle transition (Fig. 3).

### Hub gene selection and analysis

**Figure 4:**
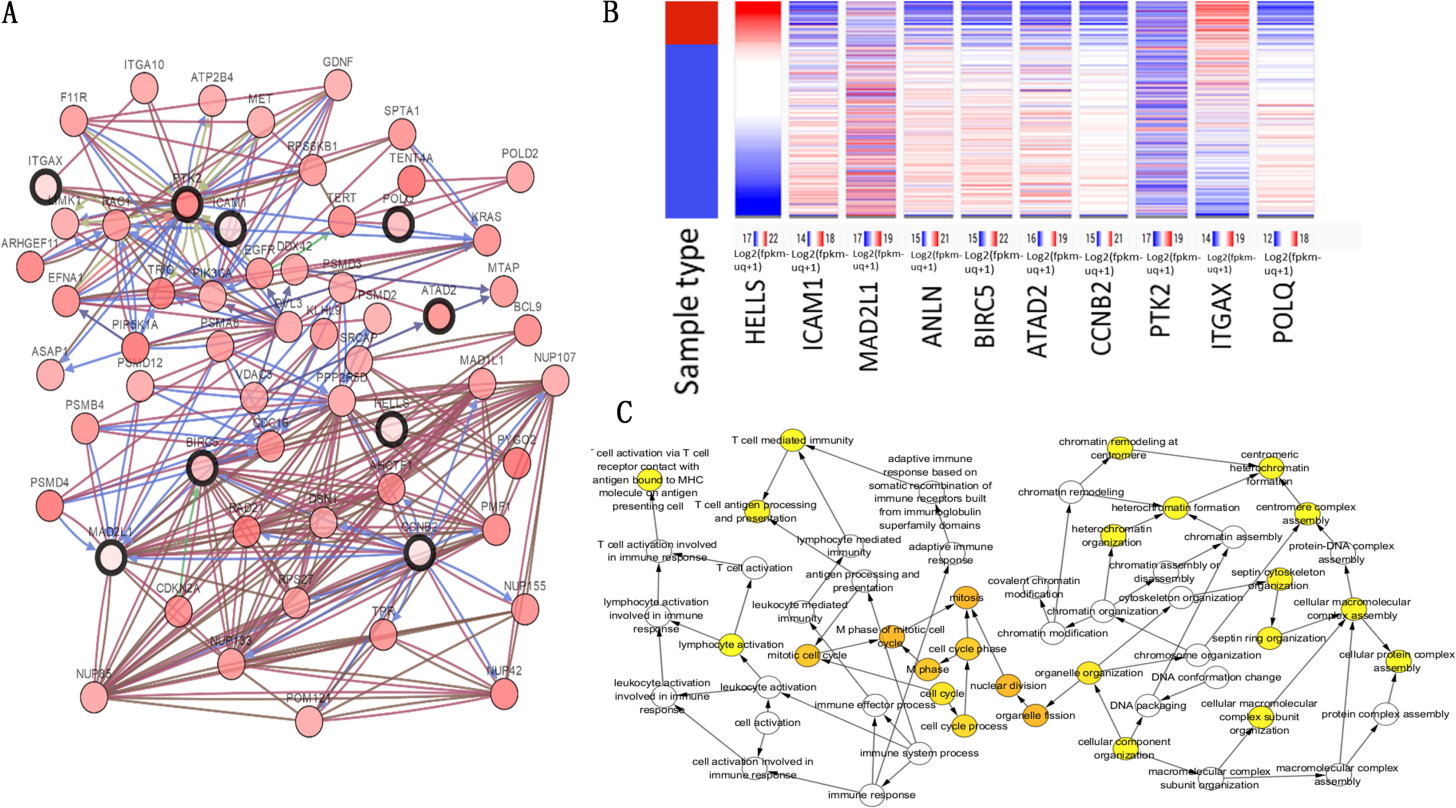
Hub gene selection and analysis. (A) Hub genes and their co-expression genes were analyzed using cBioPortal. Nodes with bold black outline represent hub genes. Nodes with thin black outline represent the co-expression genes. (B) Hierarchical clustering of hub genes was constructed using UCSC. (C) The biological process analysis of hub genes was constructed using BiNGO.

The top 10 node degree genes were MAD2L1, POLQ, HELLS, ANLN, BIRC5, ATAD2, CCNB2, PTK2, ICAM1 and ITGAX (Table 3). A network of hub genes and their co-expressed genes were analyzed using the cBioPortal online platform (Fig. 4A). The analysis of the biological processes of the hub genes, which was constructed using plug-in BiNGO, is shown in Fig. 4C. Hierarchical clustering indicated that the hub genes could differentiate cancer samples from noncancerous samples (Fig. 4B). These genes may play a significant role in lung cancer development.

**Table 3:** Functional roles of 10 hub genes.

Genes symbol	Full name	Function
MAD2L1	Mitotic arrest deficient 2 like 1	Preventing the onset of anaphase
POLQ	DNA polymerase θ	Alternative nonhomologous end joining
HELLS	Helicase, lymphoid specific	DNA strand separation
ANLN	Aniline actin binding protein	Cell growth and migration
BIRC5	Bucovina IAP repeat containing 5	Preventing apoptotic cell death
ATAD2	ATPase family AAA domain containing 2	Chaperone-like functions
CCNB2	Cyclin B2	The cell cycle regulatory machinery
PTK2	Protein tyrosine kinase 2	Cytoplasmic protein tyrosine kinase
ICAM1	Intercellular adhesion molecule 1	Endothelial cells and cells of the immune system
ITGAX	Integrin subunit alpha X	Encoding the integrin alpha X chain protein

### Association between HELLS and ICAM1 expression and prognoses in lung cancer patients

**Figure 5:**
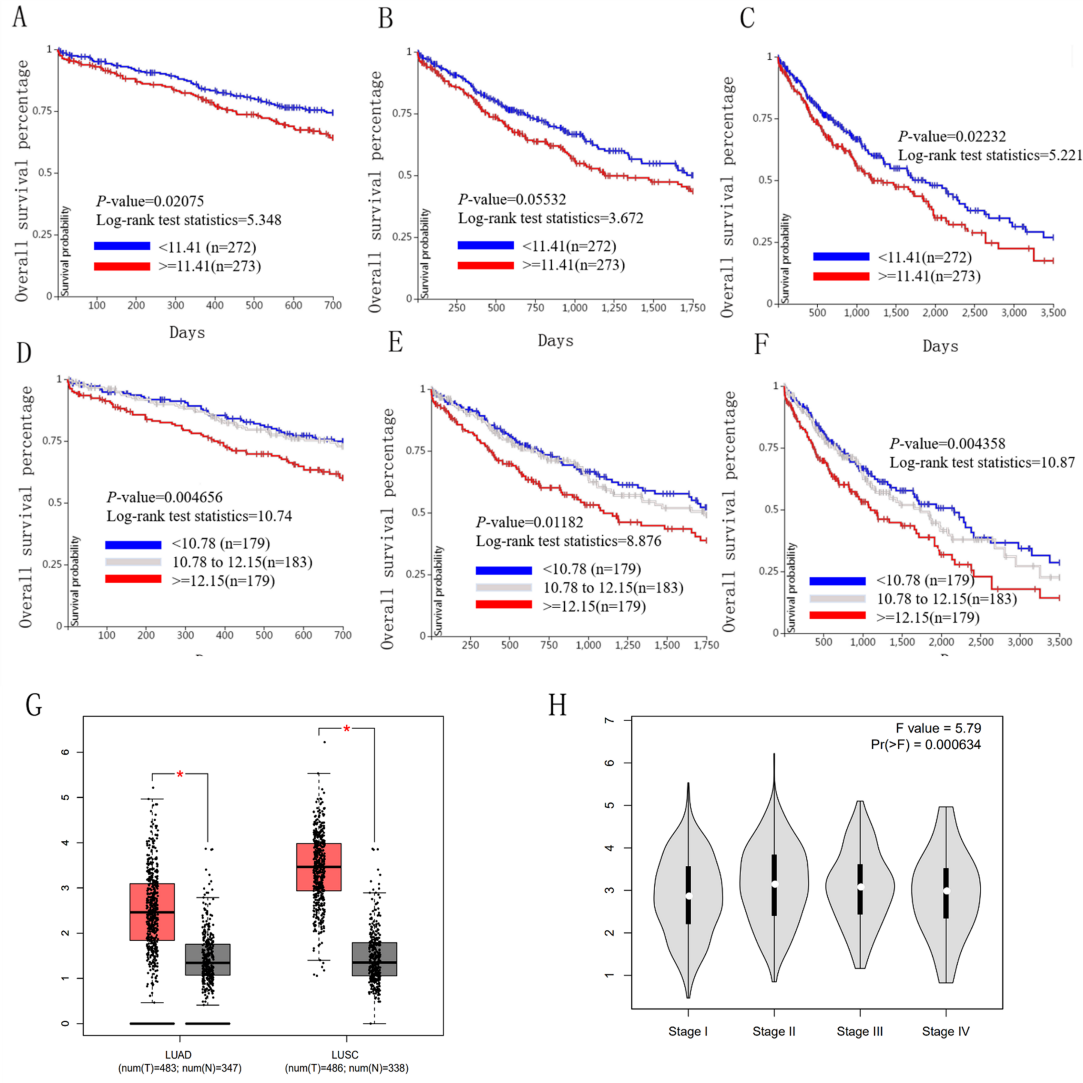
Overall survival and disease-free survival analyses of different HELLS expression lung cancer patients. (A–F) Overall survival and disease-free survival analyses of the HELLS were performed in TCGA online website. (G and H) The mRNA level of HELLS was evaluated in lung cancer using GEPIA analysis, *p* < 0.05 was considered to indicate a statistically significant diﬀerence.

**Figure 6:**
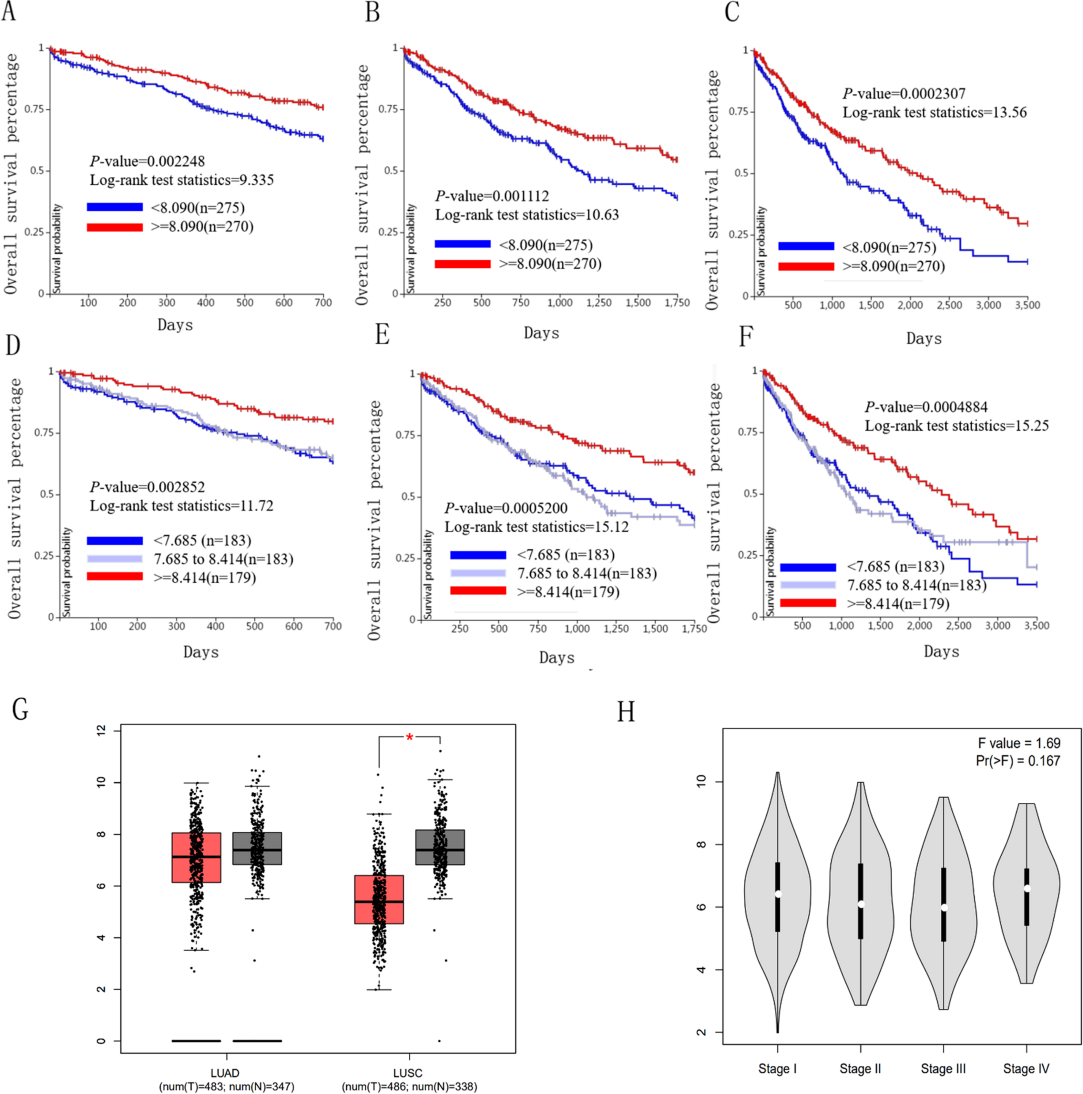
Overall survival and disease-free survival analyses of different ICAM1 expression lung cancer patients. (A–F) Overall survival and disease-free survival analyses of the ICAM1 were performed in TCGA online website. (G and H) The mRNA level of ICAM1 was evaluated in lung cancer using GEPIA analysis *p* < 0.05 was considered to indicate a statistically significant diﬀerence.

**Figure 7:**
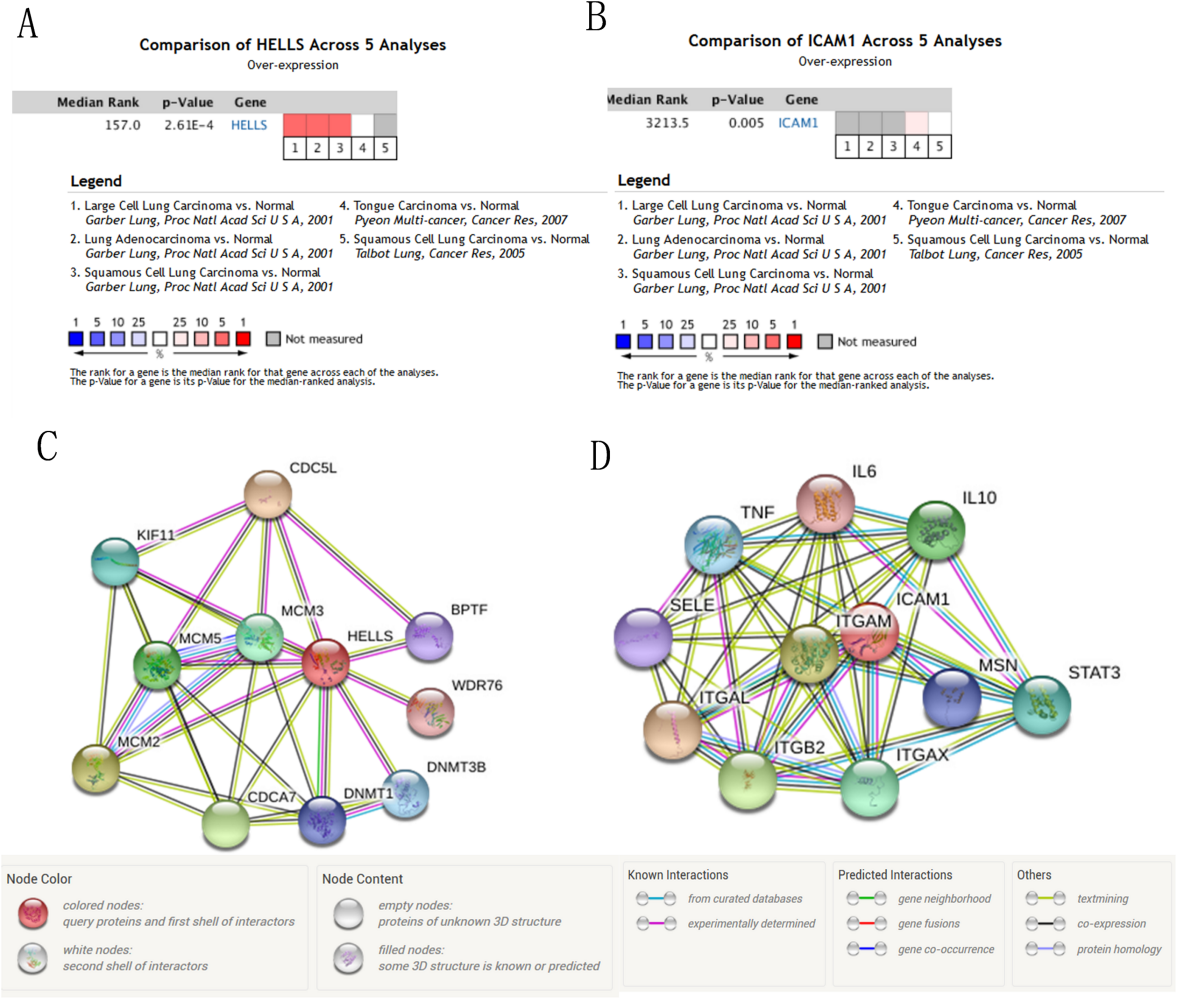
The mRNA level of HELLS and ICAM1, the PPI network of HELLS and ICAM1 were constructed. (A and B) The mRNA level of HELLS and ICAM1 were evaluated in lung cancer among four studies using ONCOMINE analysis. (C and D) PPI network of HELLS and ICAM1 were constructed by STRING database.

We used the TCGA website to further explore the 10 central genes related to survival of lung cancer patients. According to curve and logarithmic rank test analysis, the elevated level of HELLS mRNA correlated significantly with OS difference in lung cancer patients (Fig. 5). Interestingly, lower ICAM1 levels also indicated poor prognoses in lung cancer patients (Fig. 6). After assessing the mRNA levels of ICAM1 and HELLS using the Oncomine online database (https://www.oncomine.org/resource/login.html) (Rhodes et al., 2004) (Figs. 7A and 7B), it was indicated that ICAM1 was down-regulated in lung cancer across five differet studies. Furthermore, HELLS expression was upregulated in lung cancer tumors. After HELLS and ICAM1 were identified from these 10 central genes, Gene Expression Profiling interactive analysis (GEPIA; http://gepia.cancer-pku.cn/) was used to validate the selected upregulated and downregulated genes (Tang et al., 2017). The GEPIA analysis includes data from TCGA and the Genotype Tissue Expression, and provides online gene expression level analysis, survival analysis, and tumor staging analysis for 33 types of cancers, including lung adenocarcinoma (LUAD) and lung squamous cell carcinoma (LUSC). The mRNA level of HELLS was evaluated in lung cancer using GEPIA analysis, and the expression of HELLS in lung cancer tissues was significantly higher than in adjacent tissues (*p* < 0.05) (Fig. 5G). The expression of ICAM-1 in LUSC was significantly lower than in paracancer tissues (*p* < 0.05) (Fig. 6G).

**Figure 8:**
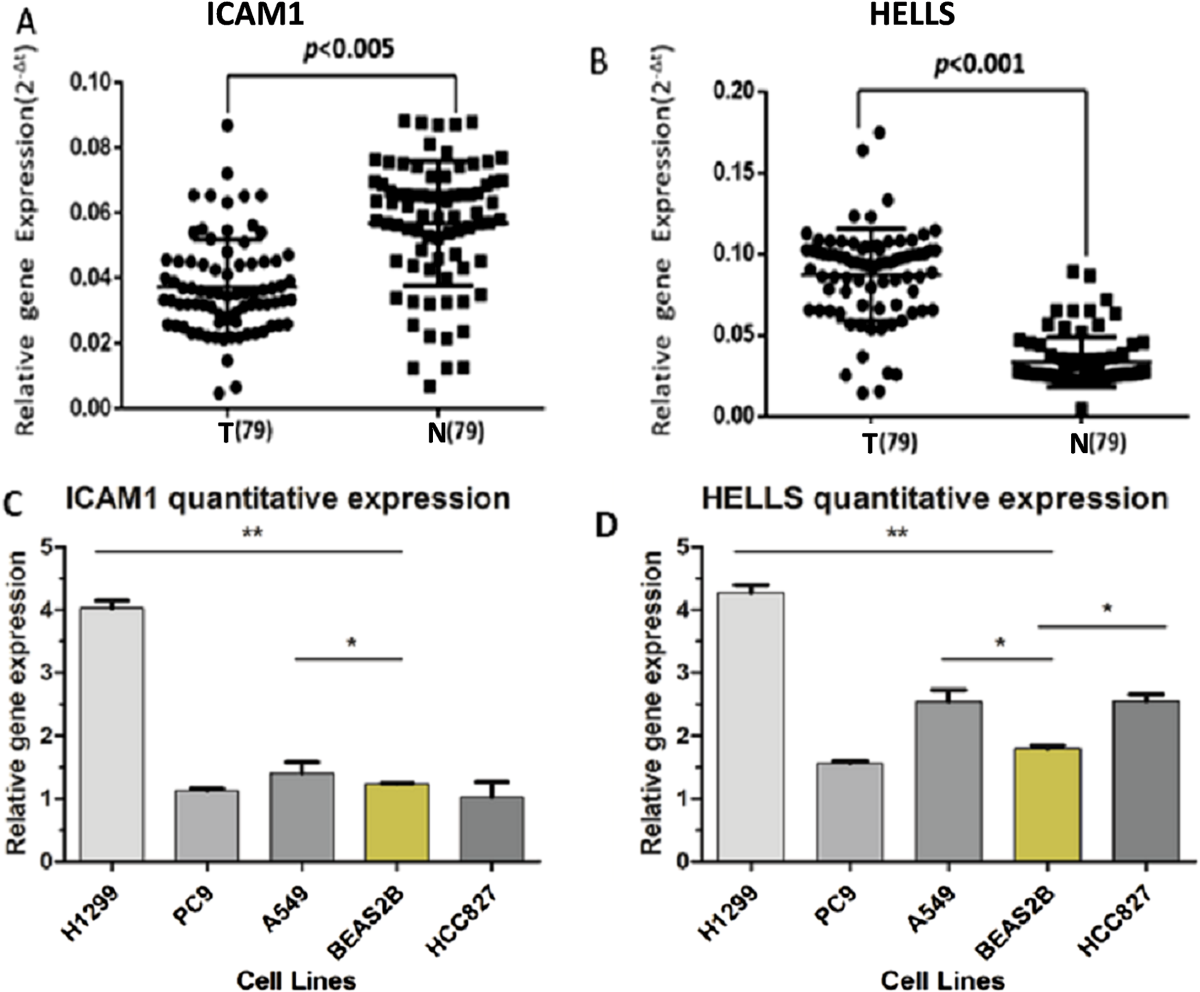
The expression levels of ICAM1 and HELLS in the lung cancer samples and the lung cancer cells. (A and B) The expression levels of ICAM1 and HELLS in the lung cancer samples. (C and D) The expression levels of ICAM1 and HELLS in the lung cancer cells.

A PPI network of ICAM1 and HELLS was constructed using the STRING database. The results indicated that HELLS was associated with other genes in the minichromosome maintenance protein family, such as MCM5, MCM3 and MCM2 (Fig. 7C). Interactions between ICAM1 and other genes associated with inflammation were also observed in the present study (Fig. 7D). To further assess the expression of ICAM1 and HELLS, we measured mRNA levels in 79 cases of lung cancer and paired paracancer samples. RT-qPCR results showed that HELLS expression in lung cancer tissues was upregulated when compared with normal tissue, while ICAM1 expression in lung cancer tissues was downregulated when compared with normal tissue. When compared with the normal lung cancer cell line BEAS2B, HELLS mRNA levels in human lung cancer cell lines H1299, A549 and HCC827 were remarkably highly expressed. However, the expression of ICAM1 only decreased in HCC827 when compared with the normal lung epithelial cell line BEAS2B (Fig. 8).

## Discussion

Over the past few decades, experts have explored the causes and underlying mechanisms of lung cancer formation and development through extensive basic and clinical research. However, most previous studies focused on the results of a single genetic event or single cohort study with inconsistent and incomplete results, and the incidence and mortality rate of lung cancer worldwide remain high (Zeng et al., 2019; Ning et al., 2019). Our study integrated three cohort profile datasets from different studies and used bioinformatics methods to perform an in-depth analysis, identifying 232 frequent changes in DEGS (40 up-regulations and 192 down-regulations). The 232 DEGs were then allocated to three groups according to GO terminology (molecular function, biological process and cell component) using a variety of methods. GO and KEGG analyses showed significant enrichment in these DEGs based on function and signaling pathway analysis. A PPI network complex was developed for the DEGs to filter the hub genes and dysregulated pathways. To determine the expression pattern, potential function, and different prognostic values of the DEGS, we performed a detailed analysis of the expression of DEGS in lung cancer patients.

HELLS (also known as SMARCA6 and PASG) is the main member of the SNF2 chromatin remodeling enzyme family. The human HELLS gene is located in the c3–d1 region of chromosome 10q23–q24, while the mouse homolog is located in the same region of chromosome 19. Related studies have shown that HELLS plays an important regulatory role during normal embryonic development (Sun et al., 2004) and encodes a lymphoid-specific helicase. Other helicases are involved in processes involving DNA strand separation, including replication, repair, recombination, and transcription. Lymphoid-specific helicase has been shown to be involved with cellular proliferation (Geiman, Durum & Muegge, 1998; Lee et al., 2000). To maintain the DNA methylation pattern of the mammalian genome (Myant & Stancheva, 2008), HELLS typically interacts with DNA methyltransferases. According to recent research, HELLS also plays an important regulatory role in cell proliferation and, possibly, in the development of cancer (He et al., 2016; Benavente et al., 2014; Teh et al., 2012; Tao et al., 2011). HELLS is a key epigenetic driver of hepatocellular carcinoma (HCC) and inhibits multiple tumor suppressor genes by promoting the occupancy of nucleosomes of NFR and enhancers to promote HCC progression (Law et al., 2019). Recent studies have shown that HELLS genes are upregulated in nasopharyngeal carcinoma, retinoblastoma, head and neck cancer, and breast cancer. However, the detailed mechanisms of HELLS in cancer, particularly the reasons for its differential expression and downstream targets, need further research. The present study screened several DEGs in three datasets to reveal that increased levels of HELLS mRNA were significantly associated with poor OS in lung cancer patients, suggesting that HELLS may be a potential novel predictor of prognosis.

Intercellular adhesion molecule 1 (ICAM1) is an important member of the immunoglobulin superfamily. It is a glycosylated transmembrane protein that plays a key role in immune synapse formation, T cell activation, leukocyte trafficking, and various cellular immune responses. A large number of studies have shown that ICAM1 shows higher expression in mesenchymal stem cells such as bone marrow, placenta, fat and periodontal ligament (Brooke et al., 2008; De Francesco et al., 2009; Sununliganon & Singhatanadgit, 2012). Studies have also shown that ICAM-1 is a marker of human and mouse liver cancer stem cells and is involved in the metastasis of liver cancer cells. Its expression is regulated by the stem cell transcription factor Nanog (Liu et al., 2013). Reduced expression of ICAM-1 could play a role in the suppression of tumor progression in many cancer cells, such as breast cancer (Ogawa et al., 1998), gastric cancer (Fujihara et al., 1999), lung cancer (Kotteas et al., 2014) and colorectal cancer (Maeda et al., 2002). Additionally, ICAM1 and CD44 may have compensatory effects to maintain the dry characteristics of esophageal squamous cell carcinoma, indicating multiple targeted therapies that can be combined and considered in cancer treatment (Tsai et al., 2015). Research has demonstrated that ICAM1 is involved in angiogenesis through the regulation of endothelial cell migration (Kevil et al., 2004). Additional studies have shown that ICAM-1 in the systemic circulation of lung cancer patients can bind to leukocyte-function associated antigen-1 of cytotoxic lymphocytes in the blood, enabling cancer cells to evade immune recognition mechanisms (Kim et al., 2017). Other studies have shown that cannabinoid-induced ICAM-1 can increase LAK cell-mediated tumor cell killing ability in lung cancer, a novel antitumor mechanism of cannabinoids (Haustein et al., 2014). From these three datasets, we identified that ICAM-1 is dysregulated in lung cancer. In combination with a series of previous studies, we found that decreased ICAM1 mRNA levels predict poor prognoses in patients with lung cancer (Melis et al., 1996; Haustein et al., 2014; Schellhorn et al., 2015). Therefore, ICAM1 may be a novel potential therapy target for lung cancer patients.

## Conclusions

In summary, we analyzed multiple cohort datasets and integrated bioinformatics to identify and screen 232 candidate genes, and we constructed a PIP network complex to screen 129 gene nodes and 10 node degree genes in DEGs. We found that elevated HELLS and decreased ICAM1 mRNA levels are predictive of poor prognoses in lung cancer patients, which could significantly improve our understanding of the causes and potential molecular events of lung cancer. However, our findings should be supplemented, and the direction for further research may include related mechanism validation studies. Whether the selected molecules have clinical significance should be verified and discussed. Therefore, further research is required to clarify the exact molecular mechanisms of these genes in lung cancer.

## Supplemental Information

10.7717/peerj.8731/supp-1 Supplemental Information 1 Verification of HELL1 and ICAM1 gene expression levels in different cell lines (standardized).

10.7717/peerj.8731/supp-2 Supplemental Information 2 We measured the level of HELLS in 79 pairs of lung cancer and adjacent tissues.

10.7717/peerj.8731/supp-3 Supplemental Information 3 The level of HELLS in 79 pairs of lung cancer and adjacent normol tissues.

## Additional Information and Declarations

### Data Availability

The following information was supplied regarding data availability: Data is available at NCBI GEO: GSE18842, GSE19188 and GSE27262. The raw data is available in the Supplemental Files.
